# Cost-effectiveness of a tailored intervention designed to increase breast cancer screening among a non-adherent population: a randomized controlled trial

**DOI:** 10.1186/1471-2458-12-760

**Published:** 2012-09-11

**Authors:** Yoshiki Ishikawa, Kei Hirai, Hiroshi Saito, Jun Fukuyoshi, Akio Yonekura, Kazuhiro Harada, Aiko Seki, Daisuke Shibuya, Yosikazu Nakamura

**Affiliations:** 1 Department of Public Health, Jichi Medical University, Shimotsuke, Tochigi, Japan; 2 Center of the Study for Communication Design & Support Office for Large-scale Education and Research Projects, Osaka University, Osaka, Japan; 3 Screening Assessment & Management Division, National Cancer Center, 5-1-1 Tsukiji, Chuo-ku, Tokyo, 104-0045, Japan; 4 Cancer Scan, Tokyo, Japan; 5 Japan Society for the Promotion of Science, Tokyo, Japan; 6 Faculty of Sport Sciences, Waseda University, Tokyo, Japan; 7 Faculty of Human Sciences, Osaka University, Osaka, Japan; 8 Cancer Detection Center, Miyagi Cancer Society, Miyagi, Japan

**Keywords:** Mammography, Tailored intervention, Cancer worry, Cost-effectiveness, Non-adherent population

## Abstract

**Background:**

Although the percentage of women who initiate breast cancer screening is rising, the rate of continued adherence is poor. The purpose of this study was to examine the effectiveness and cost-effectiveness of a tailored print intervention compared with a non-tailored print intervention for increasing the breast cancer screening rate among a non-adherent population.

**Methods:**

In total, 1859 participants aged 51–59 years (except those aged 55 years) were recruited from a Japanese urban community setting. Participants were randomly assigned to receive either a tailored print reminder (tailored intervention group) or non-tailored print reminder (non-tailored intervention group). The primary outcome was improvement in the breast cancer screening rate. The screening rates and cost-effectiveness were examined for each treatment group (tailored vs. non-tailored) and each intervention subgroup during a follow-up period of five months. All analyses followed the intention-to-treat principle.

**Results:**

The number of women who underwent a screening mammogram following the reminder was 277 (19.9%) in the tailored reminder group and 27 (5.8%) in the non-tailored reminder group. A logistic regression model revealed that the odds of a woman who received a tailored print reminder undergoing mammography was 4.02 times those of a women who had received a non-tailored print reminder (95% confidence interval, 2.67–6.06). The cost of one mammography screening increase was 2,544 JPY or 30 USD in the tailored intervention group and 4,366 JPY or 52 USD in the non-tailored intervention group.

**Conclusions:**

Providing a tailored print reminder was an effective and cost-effective strategy for improving breast cancer screening rates among non-adherent women.

## Background

Breast cancer is the leading cause of cancer death in women [1]. It is estimated that 458,400 women died of breast cancer in 2008 [1]. The 10-year survival rates for breast cancer vary dramatically according to the stage of detection; from 20% when there is distant spread of the disease to 90% when the disease is localized [2]. Therefore, the early detection of breast cancer is of great public health importance. In fact, breast cancer mortality can be reduced by 20–30% in women over 50 years old in developed countries when the screening coverage is over 70% [3].

The most effective detection method to reduce breast cancer mortality is regular screening with mammography. However, non-adherence limits the potential benefit of screening. A review of 37 studies indicated that the weighted average adherence rate was only 46.1% (95% confidence interval 39.4%–52.8%) among eligible women [4]. Therefore, to reduce breast cancer mortality through regular mammography screening, the biggest challenge is to develop cost-effective and easily implemented interventions that promote higher rates of participation among non-adherent women.

One of the effective evidence-based strategies that is often employed to increase mammography screening is the provision of tailored interventions [5,6]. Tailored interventions include an individual assessments and the provision of tailored messages through print, telephone or in-person [7]. A previous study suggested that tailored print interventions are effective and economical strategies to increase mammography screening compared with tailored telephone or in-person interventions [8]. However, few studies have examined the applicability of tailored print interventions across a range of settings and populations despite their importance.

The purpose of this study was to examine the effectiveness and cost-effectiveness of a tailored print intervention compared with a non-tailored print intervention for increasing breast cancer screening rates among a non-adherent population.

## Methods

### Setting

The study was conducted in an urban area of Japan. According to the 2009 census, the area’s population is approximately 174,600. The local government introduced an organized breast cancer screening system in 2004. Information about the breast cancer screening program is provided by the local government through its website and monthly community newsletter. During the study period the recommended screening program was a biannual mammogram and clinical breast examination for women aged 40 years or older. Mammograms and clinical breast examinations were available for a payment of 1,000 JPY (around 12 USD). The breast cancer screening was provided at the local medical association’s network of twelve clinics. Through this network, the local government provides breast cancer screening to more than 1800 individuals, or around 12.2% of the eligible population in this community each year.

### Procedure

This study used a prospective randomized controlled design in a Japanese community setting. The women selected for inclusion in the study met the following criteria: (a) had no recorded mammogram in the previous 24 months (within the organized screening program conducted by the local government), and (b) were 51–59 (except 55) years old. The women aged 55 years were excluded because the local government provided them with a free screening coupon.

**Figure 1:**
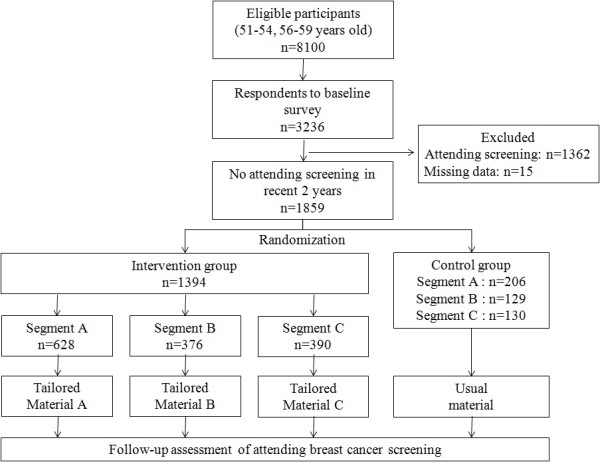
Flow diagram of the trial process.

The flow of participants is described in Figure 1. In October 2009, a total of 8100 women were identified from the local health department’s database. A baseline mail survey was conducted to obtain individual psychological assessments. The study’s aim and plan were announced on the local government’s web site and informed consent for enrollment in this study was obtained by returning the questionnaire. Of the 8100 women who received the mail survey, 3236 replied. However, 1362 women were subsequently excluded based on the eligibility criteria, and 15 were excluded because of missing data. Following the baseline survey, a total of 1859 eligible women were randomly assigned to one of two conditions: approximately 75% of the sample (n = 1394) to the tailored intervention and 25% (n = 465) to the non-tailored intervention. The ratio of the participants’ allocation between the two intervention groups was decided by the local government, because this study was conducted as part of the local government-led health promotion program. Since the local government hypothesized that the tailored intervention would be more effective than the non-tailored one, they wanted to allocate more participants to the tailored intervention group. The incentive for the local government to conduct this study was to increase the number of the participants underwent screening, so that we employed uneven allocation of the participants as suggested by the local government.

In November 2009, after the randomization, the local government mailed a print reminder to prompt study participants to participate in the mammography screening: intervention group got tailored reminder and control group got non-tailored reminder. Those who wanted to participate in the breast cancer screening program were required to send a post card to the local government to get screening tickets issued by the local government. Then, the screening applicants with a screening ticket visit one of the twelve clinics designated by the local government to receive the screening mammography. Breast cancer screening was available from November 2009 through March 2010.

### Intervention

The tailored intervention had two components: (1) individual assessment, and (2) an assessment-based tailored message.

#### Individual assessment

As a first step of tailored interventions, a variety of variables such as age, risk, and barriers to screening, as well as psychological variables based on theoretical models have been used for individual assessment. This study employed two theory-based variables: intention to undergo mammography and cancer worry.

Intention to have a mammogram is a theoretical construct from the Theory of Planned Behavior [9], whereby one of the strongest immediate determinants of behavior is a person’s intention to perform it. Empirically, the intention to undergo screening remains one of the strongest and most consistent factors associated with actual breast cancer screening [10,11]. Cancer worry is defined as an “emotional reaction to the threat of cancer” [12,13]. A 2005 review supported the importance of cancer worry in understanding cancer screening behavior [14]. Furthermore, these two variables were found to predict mammography attendance among Japanese women [15] and effectively discriminate non-adherent women.

Based on these two variables, we identified the following three segments among non-adherent women: those with high intention (segment A); those with low intention and high breast cancer worry (segment B); and those with low intention and low breast cancer worry (segment C).

#### Tailored message

Three types of tailored persuasive statements that were suited to each segment were developed through formative research by the researchers and the social marketers. Formative research is the basis for developing effective messages and materials for influencing behavior change [16-18]. It helps researchers identify and understand the characteristics, interests, behaviors and needs of target populations that influence their decisions and actions. Table 1 lists an example of each tailored persuasive statement. For women with high intention (segment A), clear information about where/when/how they could receive screening was conveyed. For women with low intention and high breast cancer worry (segment B), a gain-framed message that emphasized the benefits of getting a mammogram was conveyed. For women with low intention and low breast cancer worry (segment C), a loss-framed message that emphasized the costs of not getting a mammogram was conveyed. These gain- and loss-framed messages were developed based on the framing postulate of Prospect Theory [19]. The framing postulate states that the choice of a risky option, such as cancer screening, may depend on the way how the option is positively or negatively framed. Individuals are more likely to avoid risks when considering gains but are willing to accept risks when considering losses [19].

**Table 1:** Tailored persuasive statement examples

**Segment**		**Type of message sent**
Control Group		Usual reminder:
		“You are due for your cancer screening”
Segment A	High screening intention	Clear information about where/when/how they can receive screening
Segment B	Low screening intention/high cancer worry	Gain-framed message:
		“Detecting cancer early can lead to a higher chance of cure”
Segment C	Low screening intention/low cancer worry	Loss-Framed Message:
		“Not detecting cancer early can increase the risk of fatality”

In addition to the tailored persuasive statement, tailored intervention group received facts about breast cancer and mammography, such as the morbidity and mortality rate of breast cancer and the importance of early detection.

#### Non-tailored message

Although no tailored persuasive statement was delivered to the control group, an A4-size print reminder was delivered to inform that they were due for a screening mammography and received general information on screening procedure and breast cancer screening.

### Data collection and survey measures

#### Main outcome measure

The main outcome measure was participation in community-organized mammography within five months of receiving a print reminder. Mammogram attendance data were collected as part of the standard record keeping functions of the twelve participating healthcare facilities. Each facility sent a written notification to the local government when a mammogram had been performed. This information was then transferred to a medical history form and used to determine the number of mammograms received.

#### Variables for individual assessment

In the baseline survey, intention to have a screening mammogram and breast cancer worry were assessed in order to divide the respondents into the segments. The concept of intention was derived from the Theory of Planed Behavior [9] and measured by a single item: whether or not they intended to attend breast cancer screening in next 12 months. One item was used to assess breast cancer worry: “How much do you currently worry about getting breast cancer someday?” Both items were developed in previous study and their validity is confirmed [15]. Four items representing psychological characteristics from the Attitude Toward Breast Cancer Screening Scale [20] and the Perceived Health Competence Scale [21] were used to assess subjective norms for screening, barriers towards screening (attitude), lack of importance of getting screening (attitude), and perceived health competence.

#### Costs

To calculate the total costs of each intervention, only costs involving the implementation of the intervention program were calculated, such as individual assessment, overhead costs, and the costs of the mailed reminder including envelopes, printing and postage.

We excluded start-up costs (e.g. for the research and development of the intervention materials) so that the cost-effectiveness of the interventions were compared as if the intervention materials were already in existence. This is because we will make the final intervention materials available to other organizations for free. This cost calculation method has been used in other similar studies [8,22].

### Statistical analysis

Descriptive analyses were performed to summarize the participants’ backgrounds and psychological measurement scores. Logistic regression with the non-tailored intervention group serving as the reference group was performed to determine if mammography uptake differed between the tailored and non-tailored group during a follow-up period of five months. We also analyzed the cost-effectiveness of the interventions by dividing the cost by the number of mammograms performed. All analyses were based on intention-to-treat, and were performed using SAS 9.1.3 statistical software.

### Ethical issues

This study was approved by the Institutional Review Board (IRB) of the National Cancer Center and adopted the principles of the Declaration of Helsinki. The IRB granted exemption of a written informed consent because of the minimal risk associated with the print reminder and a guarantee of at least usual care for all eligible community members by the local government.

## Results

### Baseline characteristics of respondents

Of the 3236 respondents, 1362 had attended breast cancer screening in the previous 24 months, and 15 of them had missing data. Thus, 1377 respondents were excluded from the trial. Among the remaining 1859 respondents, 834 were in segment A, 505 were in segment B, and 520 were in segment C. They were randomly assigned to either the tailored intervention (n = 1394) or non-tailored intervention (control) group (n = 465) while adjusting for the distribution of each segment. Table 2 presents the baseline psychological characteristics of the two study groups. There were no significant differences in the scores on the psychological variables between the groups.

**Table 2:** Comparison of baseline characteristics between study groups

	**Intervention Group**		**Control Group**		
	**(n = 1394)**		**(n = 465)**		
**Psychological variable (range:1–5)**	**Mean**	**SD**	**Mean**	**SD**	***p*-value**^**a**^
Subjective norms for screening	3.07	1.35	3.04	1.38	0.629
Barriers toward screening	2.65	1.30	2.58	1.29	0.349
Barriers on screening	2.91	1.30	2.91	1.39	0.899
Lack of importance of screening	2.21	1.21	2.21	1.21	0.971
Perceived health competence	3.07	1.07	3.14	1.11	0.219
^a^ based on *t*-tests					

The psychological characteristics of the respondents in each segment are shown in Table 3. In segment A, the subjective norm score was significantly higher, and the score of barriers to screening and lack of importance of screening were significantly lower than those of the other segments. Compared with the other segments, segment B perceived more barriers towards screening. The lack of importance score was highest and the subjective norms score was lowest in segment C.

**Table 3:** Comparison of baseline characteristics between segments

	**Segment A**		**Segment B**		**Segment C**		***p*-value**^**a**^	**Post hoc test**^**b**^
	**(n = 834)**		**(n = 505)**		**(n = 520)**			
	**Mean**	**SD**	**Mean**	**SD**	**Mean**	**SD**		
Subjective norms for screening	3.46	1.35	3.07	1.29	2.38	1.20	<0.001	C < B < A
Barriers towards screening	2.52	1.30	2.85	1.31	2.47	1.23	<0.001	A,C < B
Barriers on screening	2.76	1.35	3.14	1.33	2.94	1.40	<0.001	A < B,C
Lack of importance of screening	1.69	0.92	2.25	1.09	3.02	1.27	<0.001	A < B < C
Perceived health competence	3.14	1.11	3.11	1.05	3.10	1.14	0.799	
^a^One-way analysis of variance								
^b^Tukey’s HSD								

### Effect of intervention on breast cancer screening attendance rate

**Figure 2:**
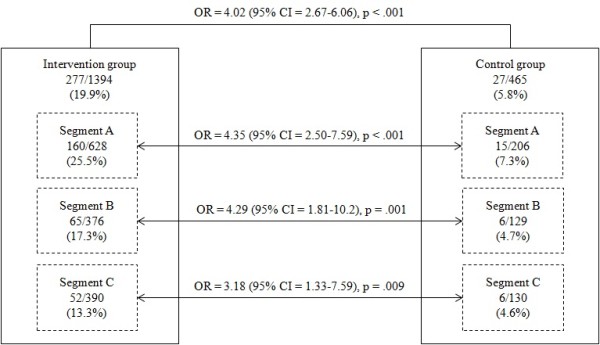
**Effect of intervention on breast cancer screening attendance.** OR = Odds Ratio, CI = Confidence Interval.

As shown in Figure 2, while only 5.8% of the non-tailored intervention group attended breast cancer screening, as high as 19.9% of the tailored intervention group attended breast cancer screening (p<0.001). In addition, the logistic regression analysis revealed that the respondents in segment A, segment B, and segment C within the tailored intervention group were more likely to attend breast cancer screening than those in each of corresponding segments in the non-tailored intervention group (Figure 2).

### Intervention costs and cost-effectiveness

Table 4 shows the specific components of each task and materials used for the interventions and the corresponding costs for each item or task. The total costs of the tailored and non-tailored interventions were 704,754 JPY or 8,390 USD (n = 1394) and 117,885 JPY or 1,403 USD (n = 465) respectively. The cost of one increase in mammography screening was 2,544 JPY or 30 USD in the tailored intervention group and 4,366 JPY or 52 USD in the non-tailored intervention group, respectively.

**Table 4:** Cost and Cost-effectiveness of tailored vs. non-tailored interventions

	**Tailored (n = 1394)**			**Non-tailored (n = 465)**		
**Item**	**Unit twice (JPY)**	**Quantity**	**Total Cost (JPY)**	**Unit twice (JPY)**	**Quantity**	**Total Cost (JPY)**
Individual Assessment						
Questionnaire	30	1,394	41, 820			0
Envelopes	42	1,394	58,548			0
Postage	175	1,394	243,950			0
Data entry and analysis	5	1,394	6,970			0
Overhead costs	10,000	9	90,000	10,000	3	30,000
Reminder						
Envelopes	26	1,394	36,244	26	*465*	12,090
Printing	43	1.394	*59,942*	43	465	*19,995*
Postage	120	1,394	167,280	120	465	55,800
Total cost			704,754			117,885
Cost per capita			506			254
Cost per extra mammograhy			2,544			4,366

*Based on administrative staff salary: 10,000JPY/day.

## Discussion

Developing cost-effective and easily implemented strategies to enhance the breast cancer screening rate among rarely screened women is of great public health importance. To our knowledge, this is the first randomized controlled study to (1) examine the effectiveness of a tailored print reminder over non-tailored print reminder, and (2) compare the cost-effectiveness of the two reminder strategies among a Japanese non-adherent population.

One of the important findings of this study was that the tailored print reminder showed not only effectiveness in increasing mammography uptake among a non-adherent population, but also cost-effectiveness over the non-tailored print reminder. Although the effectiveness and applicability of tailored interventions to promote mammography screening have been widely examined [5,6], the Japanese population is not represented in those studies. In addition, very few studies have indicated the cost-effectiveness of tailored interventions. Our results indicate that a tailored print reminder is a simple, powerful and economical method to increase mammography uptake in the community. When implementing regular community interventions, cost-effectiveness is one of the most important factors to consider. However, the implementation of this type of tailored intervention is dependent on whether the community has a call-recall system in which print reminders corresponding with each woman’s individual characteristics or attitudes are sent.

The second important finding of this study was that it showed that the psychologically-based segmentation contributed to the effectiveness of the intervention. The logistic regression analysis revealed that respondents in the tailored group were approximately four times more likely to participate in breast cancer screening than the non-tailored group. Among the psychological variables, intention to obtain a mammogram and cancer worry play important role in discriminating non-adherent women [15]. In accordance with the psychological differences between women in these three segments, we designed totally different messages based on the framing postulate of Prospect Theory [19]. This seemed to be an effective way to increase mammography attendance rates in each group. Therefore, if possible, a preliminary assessment of the psychological backgrounds of the target population, including intention and cancer worry is needed to design effective interventions.

This research has several limitations. First, this study was conducted among women living in an urban area, so the results may not be generalizable to other groups of women in different settings. Second, the amount of available data was limited, and the behavioral pathways cannot be identified. Additional data, including demographic variables, might provide information about the reasons for responding or not responding to the print reminders. Third, because the tailored intervention group received not just the tailored persuasive message based on individual assessment but a greater amount of persuasive information such as the morbidity and mortality rates of breast cancer and the importance of early detection, the difference in mammography screening rate between the groups cannot be interpreted as a result of tailoring per se. Fourth, although we calculated the total costs of the interventions, we were not able to consider the cost of building a call-recall system. This might limit the implementation of our method to communities without call-recall systems.

## Conclusions

The tailored print reminder, which considered the psychological backgrounds of the target population, seemed to increase mammography attendance rates among a Japanese non-adherent population. Extrapolation to expected nationwide rates shows that there is a potential to reduce breast cancer mortality through increased screening.

## Competing interest

The authors declare that they have no competing interests.

## Authors' contributions

YI and KH conceived the study, performed the data analysis, drafted and revised the manuscript. HS conceived the study and participated in the interpretation of the results and discussions for manuscript writing and finalization. JF and AY conceived the study and collected the data and participated in the interpretation of the results. KH performed the data analysis and participated in the interpretation of the results and discussions for manuscript writing and finalization. AS and DS contributed in the discussions for conceptualizing the study design and analysis methods. YN participated in the interpretation of the results and discussions for manuscript writing and finalization. All authors read and approved the final manuscript.

## Pre-publication history

The pre-publication history for this paper can be accessed here:

http://www.biomedcentral.com/1471-2458/12/760/prepub
