# Correction to: Cost-effectiveness of a tailored intervention designed to increase breast cancer screening among a non-adherent population: a randomized controlled trial

**DOI:** 10.1186/s12889-020-09822-y

**Published:** 2020-11-16

**Authors:** Yoshiki Ishikawa, Kei Hirai, Hiroshi Saito, Jun Fukuyoshi, Akio Yonekura, Kazuhiro Harada, Aiko Seki, Daisuke Shibuya, Yosikazu Nakamura

**Affiliations:** 1 Department of Public Health, Jichi Medical University, Shimotsuke, Tochigi Japan; 2 Center of the Study for Communication Design & Support Office for Large-scale Education and Research Projects, Osaka University, Osaka, Japan; 3 Screening Assessment & Management Division, National Cancer Center, 5-1-1 Tsukiji, Chuo-ku, Tokyo, 104-0045 Japan; 4 Cancer Scan, Tokyo, Japan; 5 Japan Society for the Promotion of Science, Tokyo, Japan; 6 Faculty of Sport Sciences, Waseda University, Tokyo, Japan; 7 Faculty of Human Sciences, Osaka University, Osaka, Japan; 8 Cancer Detection Center, Miyagi Cancer Society, Miyagi, Japan

**Correction to: BMC Public Health 12, 760 (2012)**

**https://doi.org/10.1186/1471-2458-12-760**

It was highlighted that in the original article [1] the Competing interests section was incorrect. This Correction article shows the incorrect and correct Competing interests section.

**Incorrect**

The authors declare that they have no competing interests.

**Correct**

Jun Fukuyoshi and Akio Yonekura are the founders of Cancer Scan Co. Ltd. Yoshiki Ishikawa a director at Cancer Scan Co. Ltd. The other authors declare that they have no competing interests.
